# Biomechanical study of different fixation constructs for anterior column and posterior hemi-transverse acetabular fractures: a finite element analysis

**DOI:** 10.1186/s13018-023-03715-7

**Published:** 2023-04-11

**Authors:** Kaifang Chen, Guixiong Huang, Yizhou Wan, Sheng Yao, Yanlin Su, Lianxin Li, Xiaodong Guo

**Affiliations:** 1 Department of Orthopaedics, Union Hospital, Tongji Medical College, Huazhong University of Science and Technology, Jiefang Avenue No.1277, Wuhan, Hubei 430022 People’s Republic of China; 2 Department of Orthopaedics, Shandong Provincial Hospital Affiliated to Shandong University, Jinan, People’s Republic of China

**Keywords:** Acetabular fractures, Finite element, Biomechanics, Quadrilateral surface, Infrapectineal plate

## Abstract

**Background:**

To compare the biomechanical properties and stability, using a finite element model, of four fixation constructs used for the treatment of anterior column and posterior hemi-transverse (ACPHT) acetabular fractures under two physiological loading conditions (standing and sitting).

**Methods:**

A finite element model simulating ACPHT acetabular fractures was created for four different scenarios: a suprapectineal plate combined with posterior column and infra-acetabular screws (SP-PS-IS); an infrapectineal plate combined with posterior column and infra-acetabular screws (IP-PS-IS); a special infrapectineal quadrilateral surface buttress plate (IQP); and a suprapectineal plate combined with a posterior column plate (SP-PP). Three-dimensional finite element stress analysis was performed on these models with a load of 700 N in standing and sitting positions. Biomechanical stress distributions and fracture displacements were analysed and compared between these fixation techniques.

**Results:**

In models simulating the standing position, high displacements and stress distributions were observed at the infra-acetabulum regions. The degree of these fracture displacements was low in the IQP (0.078 mm), as compared to either the IP-PS-IS (0.079 mm) or the SP & PP (0.413 mm) fixation constructs. However, the IP-PS-IS fixation construct had the highest effective stiffness. In models simulating the sitting position, high fracture displacements and stress distributions were observed at the regions of the anterior and posterior columns. The degree of these fracture displacements was low in the SP-PS-IS (0.101 mm), as compared to the IP-PS-IS (0.109 mm) and the SP-PP (0.196 mm) fixation constructs.

**Conclusion:**

In both standing and sitting positions, the stability and stiffness index were comparable between the IQP, SP-PS-IS, and IP-PS-IS. These 3 fixation constructs had smaller fracture displacements than the SP-PP construct. The stress concentrations at the regions of quadrilateral surface and infra-acetabulum suggest that the buttressing fixation of quadrilateral plate was required for ACPHT fractures.

## Background

Acetabular fractures have become a common phenomenon, and its surgical treatment has always been a challenge for orthopaedic trauma surgeons [1]. The gold standard treatment consists of early anatomical reduction and adequate internal fixation, thereby allowing for effortless mobility and early recovery [2, 3].

The anterior column and posterior hemi-transverse (ACPHT) fracture pattern constitute approximately 20% of all acetabular fractures in the elderly [2, 4, 5]. This type of fracture affects both the anterior and posterior columns of the acetabulum and has been traditional treated with a combined approach of column plates and lag screw fixation techniques [6]. However, the choices of internal fixation technique for ACPHT fracture remain controversial [7–10]. Yildirim et al. [11] tested a total of five different fixation techniques under two loading conditions (standing and sitting) by using a finite element (FE) model. The study concluded that the two-column plate fixation technique was not required for acetabular transverse fracture. Similar studies have not been performed on the modality of the ACPHT fracture treatment. Meanwhile, different anterior intra-pelvic surgical approaches such as modified Stoppa [12, 13], pararectus [2, 14], and supra-ilioinguinal [15] have led to the development of two different anterior column plate fixation techniques: the suprapectineal and infrapectineal fixation [16, 17]. However, it remains elusive as to which of these two techniques is better at treating ACPHT fractures.

Additionally, it remains controversial as to whether the posterior column and quadrilateral surface fractures should be treated with periarticular lag screws or quadrilateral surface buttress plate [18–20]. A special infrapectineal quadrilateral surface buttress plate (IQP) was developed to treat patients with two-column acetabular fractures. The IQP achieved exceptionally good results in clinical settings. However, no comparative biomechanical data between the newly developed quadrilateral surface buttress plate and traditional column plate with lag screws are available.

As such, we designed this study for biomechanical and stability comparison, using an FE model, of four fixation constructs for the treatment of ACPHT acetabular fractures under two physiological loading conditions (standing and sitting).

## Methods

### Generation of a 3D model

**Fig. 1:**
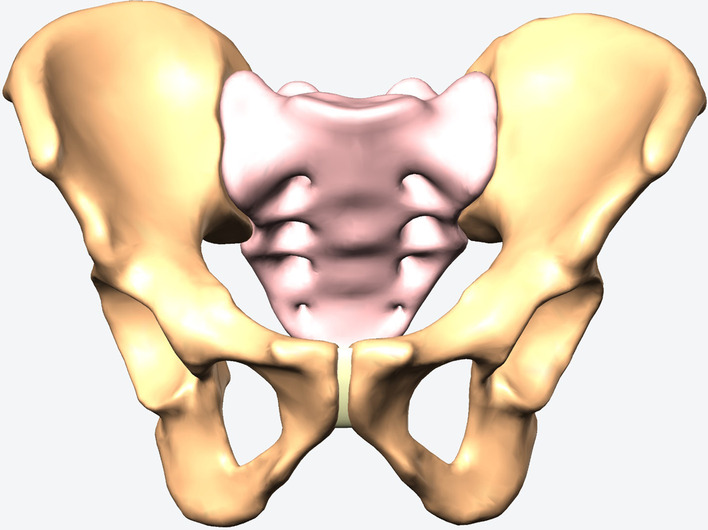
Standard model of pelvis with a cortical shell of 1.6 mm thickness wrapped around the cancellous core

The Ethics Committee of Tongji Medical College, Huazhong University of Science and Technology, gave a final approval for this study. A 3D model of the pelvis was constructed from computed tomography scan images (0.625 mm slice thickness) of a healthy male (age 60 years, height 172 cm, and body weight 70 kg) by an image processing software (Mimics 17.0, Materialize, Belgian). The model was exported in stl format for editing and optimisation with the reverse engineering software Geomagic Studio, 2012 (Raindrop, USA). The model was further standardised by using the migration function with a cortical shell of 1.6 mm thickness wrapped around the cancellous core (Fig. 1).

### Generation of fracture and fixation models

**Fig. 2:**
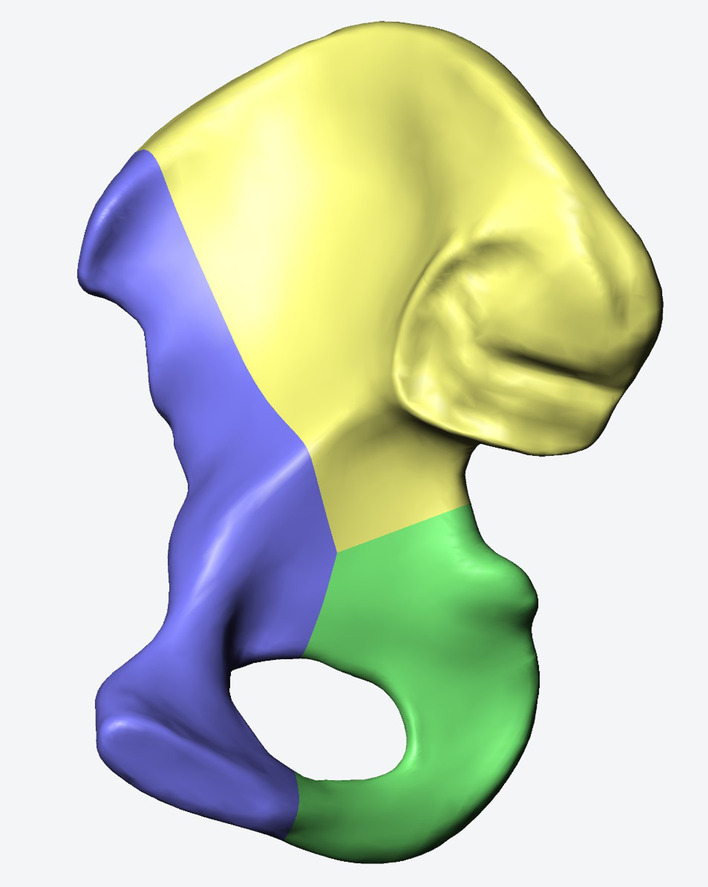
Generation of a standard ACPHT fracture model. The hemi-pelvis was divided into three bone fragments, anterior column (blue), posterior column (green), and roof column (yellow), by two fracture lines of anterior column and posterior column

**Fig. 3:**
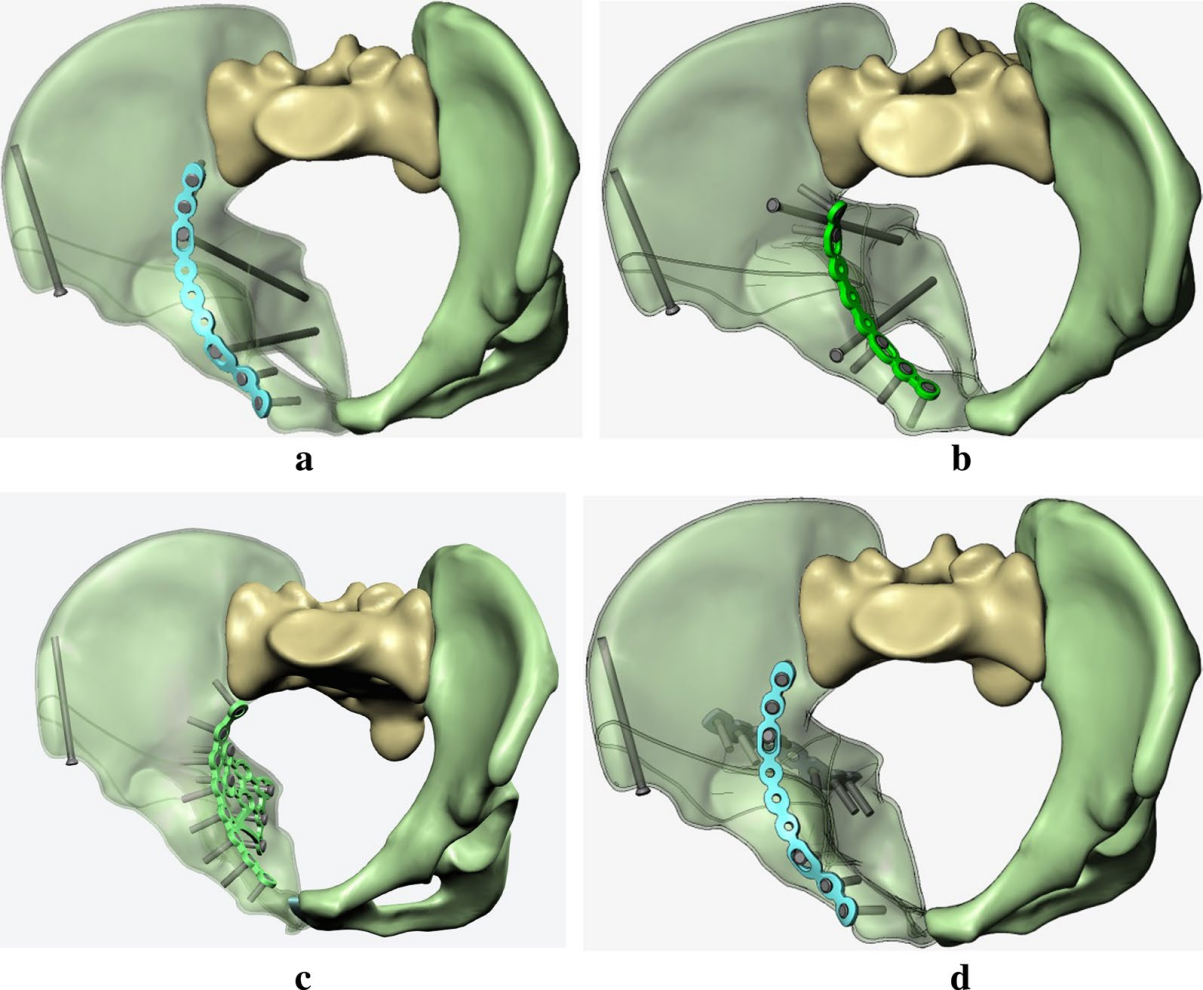
3D models of four different fixation systems: **a** a suprapectineal plate combined with posterior column and infra-acetabular screws (SP-PS-IS); **b** an infrapectineal plate combined with posterior column and infra-acetabular screws (IP-PS-IS); **c** a special infrapectineal quadrilateral surface buttress plate (IQP); and **d** a suprapectineal plate combined with a posterior column plate (SP-PP)

The 3D model was segmented with Solidworks, 2017 (Dassault, France), to create a general ACPHT fracture model (Fig. 2). Using similar technique, four different kinds of internal fixation models were also constructed and then assembled with the ACPHT fracture model to generate 3D models of ACPHT fractures that are used with their corresponding fixation techniques (Fig. 3a–d). A lag screw was implanted at the anterior iliac spine to ensure clinical relevance of internal fixation techniques.

### Construction of a standardised FE model

The standard pelvic model was analysed using Abaqus, 6.14 (Simulia, Dassarult, USA) to generate a standardised FE model. The cortical, cancellous, plate, and screw materials were set to be isotropic. The material properties of all screws and plates used in this study are titanium alloys. The contact between the bone fragments was set to surface contact; the tangent behaviour was hard contact, and the contact property was set to penalty friction with a coefficient of 0.30. The contact property between bone and plate was surface contact. The tangent behaviour was hard contact, and the contact property was penalty friction, with a coefficient of 0.45. The contact relationship between the plate and screws was set as a binding relationship. Six degrees of freedom of the sacroiliac joint and the pubic symphysis were fixed [21].

**Fig. 4:**
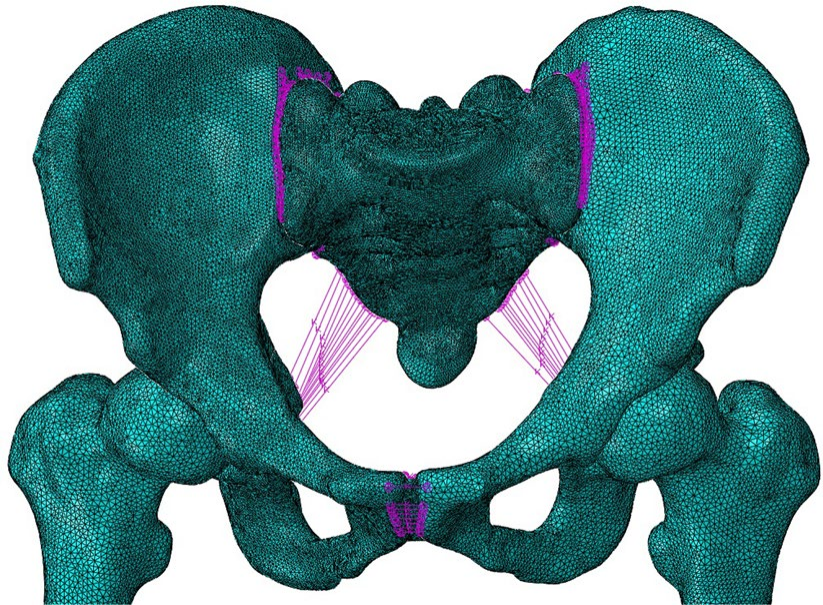
FE model of the pelvis

The ligaments and other ancillary structures were defined according to their corresponding anatomical positions on the solid surface model. The sacrospinous, sacrotuberous, anterior sacroiliac, posterior sacroiliac, sacroiliac interosseous, superior pubic, and arcuate pubic ligaments were defined in the standardised FE model. The number of springs, modulus of elasticity, and Poisson's ratio of each component were set according to previous studies [22, 23] (Tables 1 and 2). Collectively, these activities resulted in the establishment of a standard pelvic FE model (Fig. 4).

**Table 1:** Material properties of each component of the pelvic model

Material	Elasticity modulus (MPa)	Poisson’s ratio
Cortical bone	18,000	0.3
Cancellous bone	150	0.2
Plates	105,000	0.3
Screws	105,000	0.3

**Table 2:** Modelling parameters of the pelvic main ligaments

Material	K value(N/m)	Number of springs
Sacrospinous	1400	10
Sacrotuberous	1500	15
Anterior sacroiliac	700	27
Posterior sacroiliac	1400	15
Sacroiliac interosseous	2800	8
Superior pubic	500	24
Arcuate pubic	500	24

### Validation of the standard FE model

**Fig. 5:**
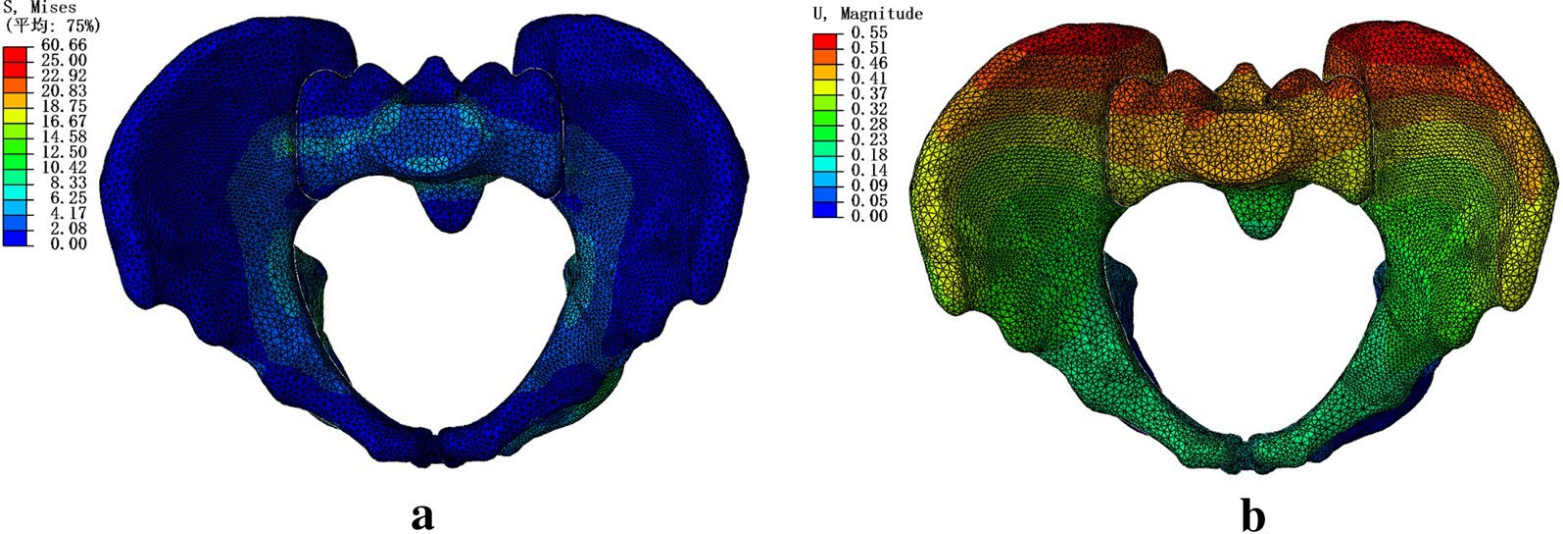
Stress **a** and displacement **b** distribution in the standard FE model of the pelvis

The pelvic model experienced a vertical force loaded on the upper surface of the sacral bone. Both the distribution of the von Mises stress and magnitudes of displacement in the standard model are shown in Fig. 5. The maximum displacement was identified at the posterior superior iliac spine, and the stress was primarily concentrated at the sacroiliac joint, superior rim, and ilium superior of the acetabulum. These data are consistent with the other experimental results [22–24] and validate that our FE model is effective and reliable.

### Loading and stress analyses

Three-dimensional models of ACPHT fractures treated with four different fixation techniques were simulated and analysed in the same way the standard FE model was validated. In this study, loading was applied in two different positions—standing and sitting.

#### Loading in the standing position

Biomechanical simulation of the pelvis in the standing position. The upper end plate of S1 vertebral body was fixed and restrained its motion at six degrees of freedom, and a total of 700 N load, representing an average body weight, was applied uniformly to the bilateral acetabulum. We used the following experimental assumptions. The mechanical properties of materials involved in the experiment remained be homogeneous and isotropic. Each element of the model had sufficient stability under force, irrespective of any deformation observed under force.

#### Loading in the sitting position

Biomechanical simulation of the pelvis in the sitting position. The bilateral ischial tubercles were fixed and restrained its motion at six degrees of freedom, and a total of 700 N load was applied to the geometric centre of the S1 end plate vertebral body to simulate the direction of force in the sitting position. The experimental assumptions remained the same as above.

## Results

The total displacement and effective stiffness of four different fixation constructs in standing and sitting positions are shown in Table 3. In the model simulating standing position, the IP-PS-IS fixation construct showed the low displacement (0.12 mm), as compared to the IQP fixation construct (0.17 mm) and the SP & PP construct (1.72 mm). Hence, the IP-PS-IS fixation construct had the highest rigidity in standing position. However, the SP & PP fixation construct was the most stable among all models simulating the sitting position. These findings are similar to those obtained from other biomechanical tests [1, 7, 16].

**Table 3:** Total displacement and effective stiffness of 4 different fixation systems in standing and sitting positions

	SP-PS-IS	IP-PS-IS	IQP	SP-PP
	Standing/sitting	Standing/sitting	Standing/sitting	Standing/sitting
Total displacement(mm)	0.33/0.48	0.12/0.47	0.17/0.52	1.72/0.44
Final stiffness(N/mm)	2121/1458	5833/1489	4117/1346	406/1590

**Fig. 6:**
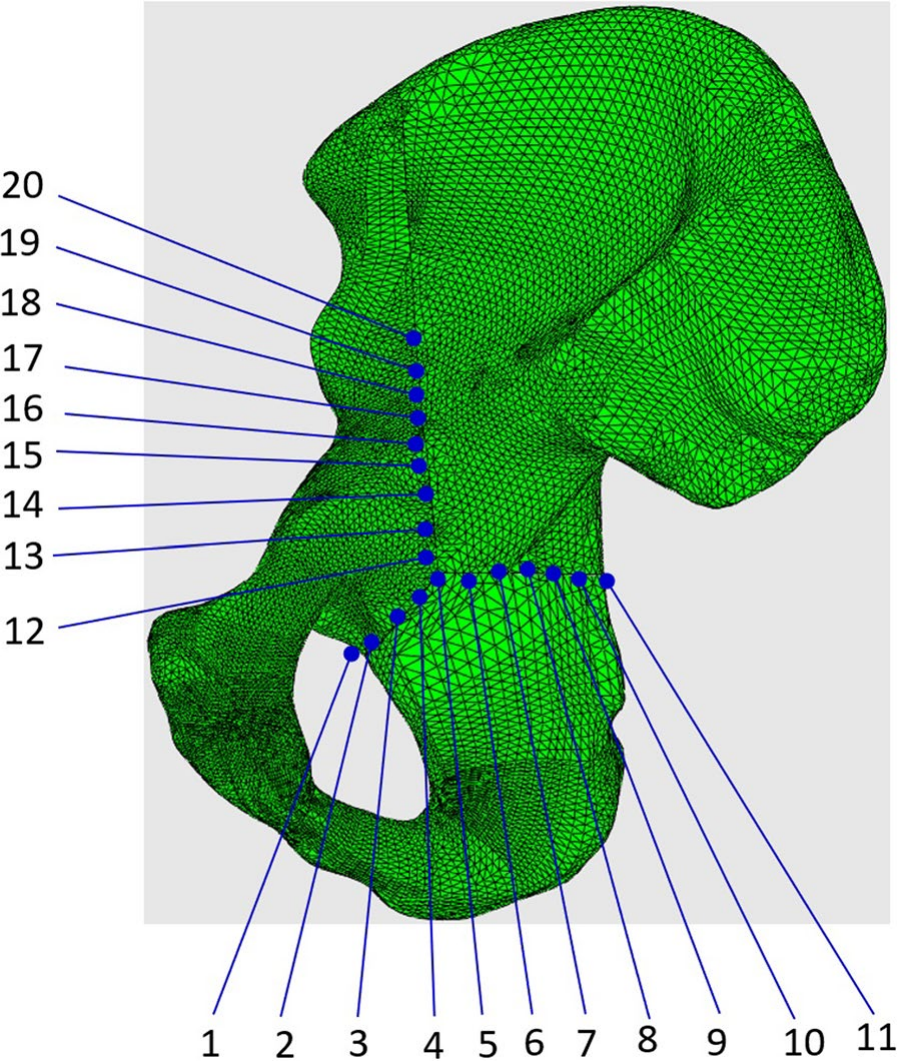
Three paths and total 20 predetermined points were obtained along the fracture line: the first path ran along the fracture line of infra-acetabulum (points 1–5); the second path ran along the fracture line of posterior column (points 6–11); and the third path ran along the fracture line of anterior column (points 12–20)

**Fig. 7:**
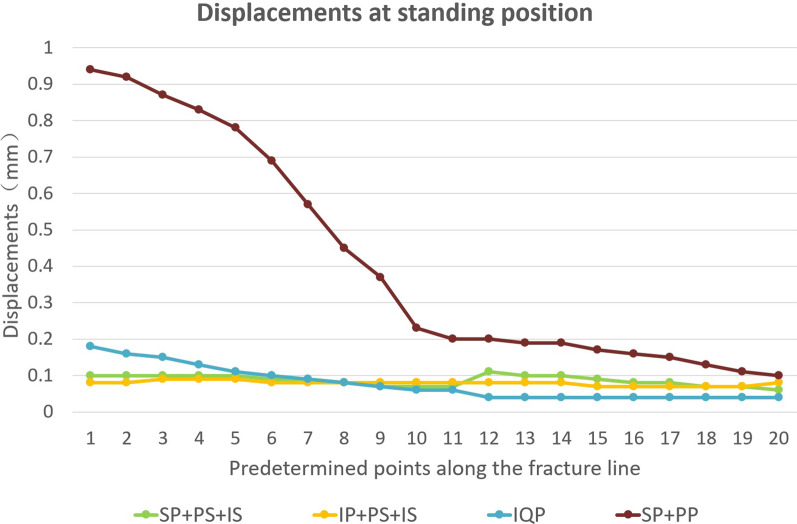
Displacements of all predetermined points along the paths in different fixation models at standing positions

**Fig. 8:**
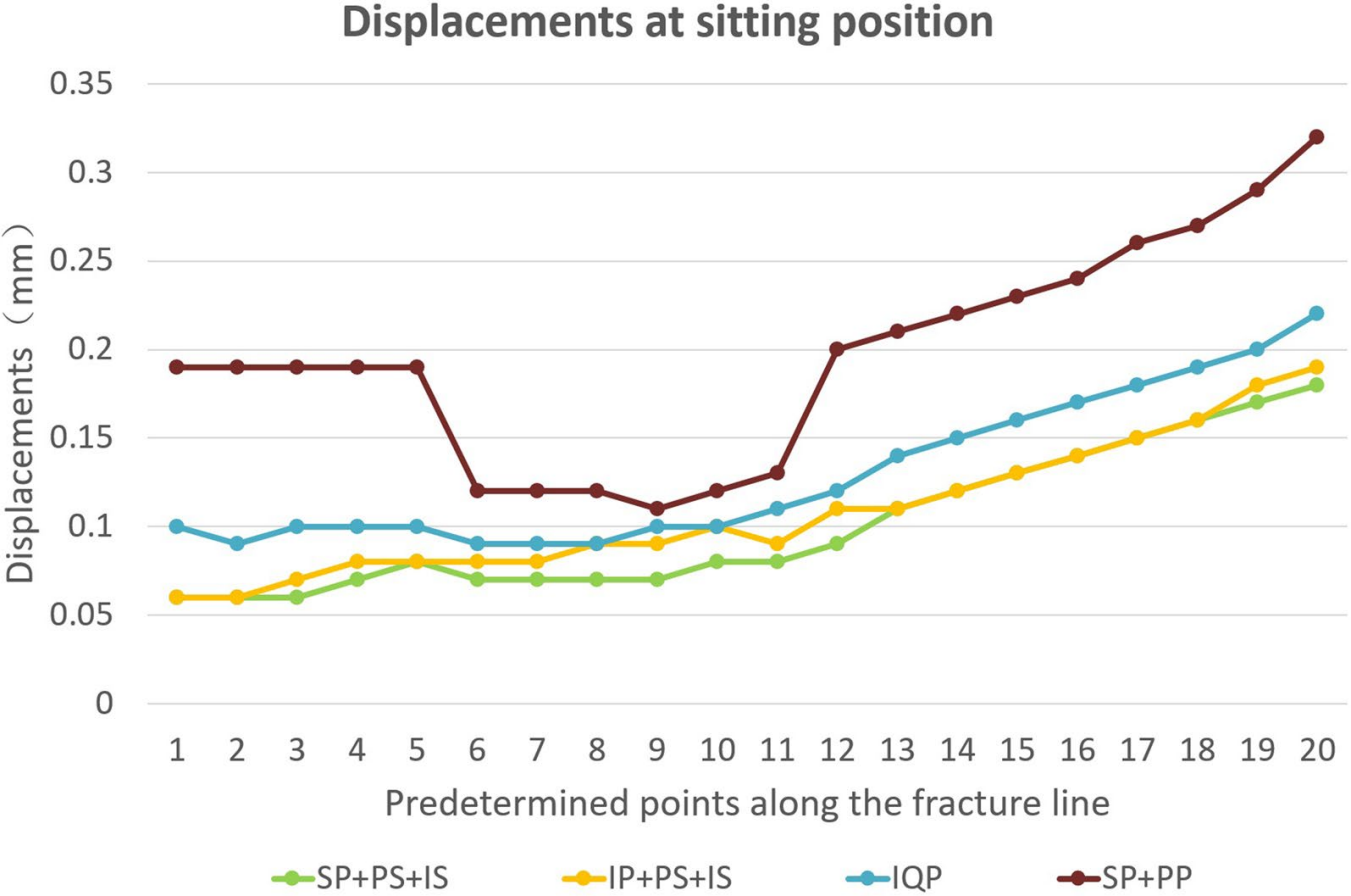
Displacements of all predetermined points along the paths in different fixation models at sitting positions

**Fig. 9:**
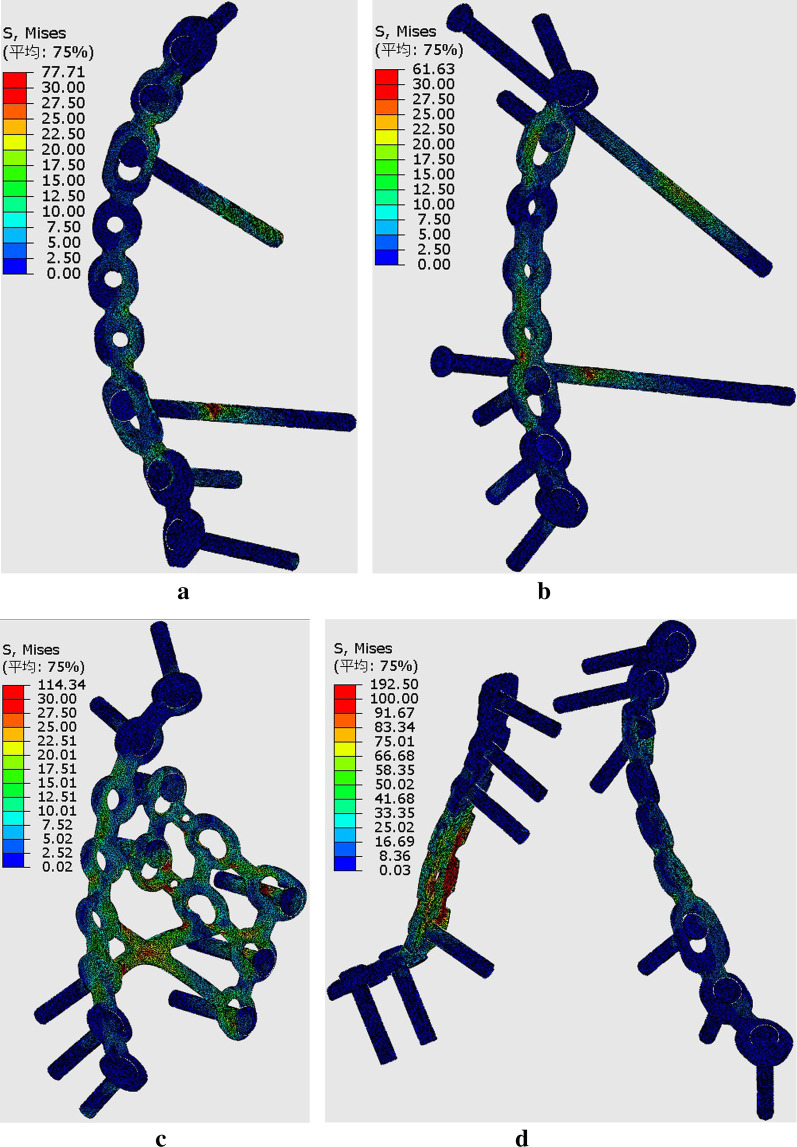
Stress distribution in different fixation constructs at standing position: **a** SP-PS-IS; **b** IP-PS-IS; **c** IQP; **d** SP-PP

To further study the displacement at the articular surface of the acetabulum, three paths and total 20 predetermined points were obtained along the fracture line (Fig. 6). The first, second, and third paths ran along the fracture lines of the infra-acetabulum (points 1–5), the posterior column (points 6–11), and the anterior column (points 12–20), respectively. The distance between each point was 5 mm. Figures 7 and 8 depict the displacements of all predetermined points along the paths in different fixation models at standing and sitting positions, respectively. The average displacement of all points along each fracture path at standing and sitting positions is shown in Table 4. In models simulating the standing position, higher displacements and stress distributions were observed at the regions of infra-acetabulum and quadrilateral surface, while stress concentration was primarily observed in the infra-acetabular screw or quadrilateral surface buttress plate (Fig. 9a–d). The degree of fracture displacements was low in the IQP (0.078 mm), as compared to the IP-PS-IS (0.079 mm) and the SP-PP (0.413 mm) fixation constructs.

**Table 4:** Average displacement of all points along each fracture path in standing and sitting positions (mm)

	Infra-acetabulum	Post. column	Ante. column	Total
	Standing/sitting	Standing/sitting	Standing/sitting	Standing/sitting
SP-PS-IS	0.1/0.066	0.078/0.073	0.084/0.139	0.087/0.101
IP-PS-IS	0.086/0.07	0.08/0.088	0.074/0.143	0.079/0.109
IQP	0.146/0.098	0.077/0.097	0.04/0.17	0.078/0.13
SP-PP	0.868/0.19	0.418/0.12	0.156/0.249	0.413/0.196

**Fig. 10:**
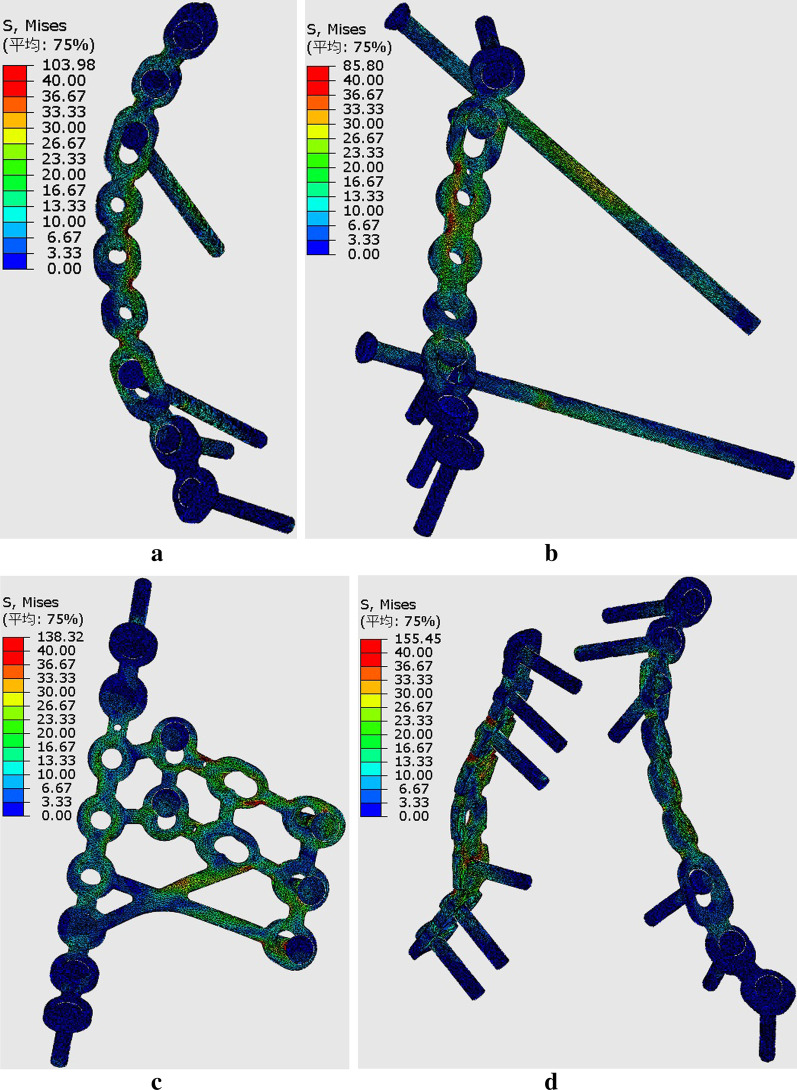
Stress distribution in different fixation constructs at sitting position: **a** SP-PS-IS; **b** IP-PS-IS; **c** IQP; **d** SP-PP

In models simulating the sitting position, higher displacements were observed at the regions of anterior column and infra-acetabulum, and the stress concentration was correspondingly observed at the anterior column plate (suprapectineal or infrapectineal) and infra-acetabular screw (Fig. 10a–d). The degree of fracture displacements was least in the SP-PS-IS (0.101 mm), followed by the IP-PS-IS (0.109 mm) and the SP-PP (0.196 mm) fixation constructs.

## Discussion

ACPHT fracture pattern is the most common type of acetabular fracture in elderly adults [2, 4, 5]. Such acetabular fractures, involving the anterior and posterior columns, are conventionally treated with two different surgical methods. One of these methods is the traditional two-column plate fixation technique through the anterior and posterior combined approaches, while the other one is the anterior column plate combined with periarticular lag screws (infra-acetabular and posterior column screws) fixation technique that uses a single classical ilioinguinal approach [25, 26]. Compared to the single anterior approach, the combined approaches have greater surgical invasiveness. However, the biomechanical comparisons between the two internal fixation methods are rarely reported.

Advancement in the anterior intra-pelvic fixation technique has resulted in the origin of the modified Stoppa [12, 13], pararectus [2, 14], and supra-ilioinguinal [15] approaches. In contrast with the classical ilioinguinal approach, these anterior intrapelvic approaches allow direct access to the QLS and enable direct reduction and buttressing of the QLS [2]. Recently, many novel internal fixation techniques have emerged. These include the infrapectineal plate and QLS buttress plate fixation techniques [17–20]. Using an FE simulation, Mehmet YÜCENS et al. [16] reported a comparative biomechanical analysis of the suprapectineal and infrapectineal fixation techniques used for analysing acetabular anterior column fractures. However, no comparative biomechanical data on these two fixation techniques used for analysing ACPHT fracture are currently available.

Hence, in this study, we compared the biomechanical effectiveness of four different fixation constructs (SP-PS- IS, IP-PS-IS, IQP and SP-PP) for the stabilisation of ACPHT fractures through FE simulation. We focused on obtaining comparative biomechanical data points such as effective stiffness, stress distributions and fracture displacements for the suprapectineal, the infrapectineal, the periacetabular lag screws, the QLS buttress plate, and the traditional two-column plate fixation techniques. All these analyses were performed in two different loading conditions—sitting and standing positions.

In the model simulating the standing position, the results of this study indicate that an infrapectineal plate with the posterior column and infra-acetabular screws (IP-PS-IS) or a special infrapectineal quadrilateral surface buttress plate (IQP) fixations techniques are superior than a suprapectineal plate with posterior column and infra-acetabular screws (SP-PS-IS) or two columns plate (SP-PP) fixation techniques. IQP plating showed least fracture displacement along the path of the anterior and posterior columns. In addition, we also found that fracture displacements in the infra-acetabulum were greater than those in the anterior and posterior columns. Higher stress concentrations were primarily observed at the infra-acetabular screw or quadrilateral surface buttress plate constructs. This suggests that it is necessary to use infra-acetabular screw or quadrilateral surface buttress plate in treating acetabular fractures with anterior and posterior column separation. This observation is in line with previously published data [8, 27].

In the model that simulates the sitting position, there is no significant difference in the effective stiffness between these four fixation constructs. A suprapectineal plate combined with posterior column and infra-acetabular screws (SP-PS-IS) fixation technique showed the least fracture displacement, followed by the IP-PS-IS technique. Interestingly, the fracture displacements at the posterior column were smaller than those at the anterior column and the infra-acetabular regions, although the ischial tubercle bears the load in the sitting position. The stress distribution was primarily concentrated on the anterior column plate (suprapectineal or infrapectineal).

The conventional two columns plate fixation technique (SP-PP) showed the highest fracture displacements in both standing and sitting positions. This may be due to the fact that, in contrast with other fixation techniques, the two plates of SP-PP fixation technique work independent of one another, while the other fixation techniques work as a unit.

There are limitations in this study. Our study is based on computer-generated simulations and not mechanical experiments. Thus, the imperial evidence is not robust. Furthermore, the bone mineral density factor was not taken into consideration while establishing different simulations. The ACPHT fracture model used in this study is more common in elderly adults with osteoporosis, which could have contributed to large fracture displacements. As such, further biomechanical studies on artificial or cadaveric pelvis are required to validate these findings.

## Conclusion

The observed stability and stiffness of the special infrapectineal quadrilateral surface buttress plate (IQP), infrapectineal or suprapectineal column plate combined with the posterior column and infra-acetabular screws fixation constructs were comparable in both standing and sitting positions. These 3 fixation constructs showed smaller fracture displacements than the two columns plate construct. Thus, the conventional two columns plate fixation technique was not required. The stress concentrations were primarily observed at the quadrilateral surface and infra-acetabulum regions, suggesting that buttressing fixation by the quadrilateral surface plate or infra-acetabular screw constructs was required for treating ACPHT fractures.

## Data Availability

The data used to support the findings of this study are available from the corresponding author upon request.

## Abbreviations

ACPHT: Anterior column and posterior hemi-transverse
FE: Finite element
QLS: Quadrilateral surface
IQP: Infrapectineal quadrilateral surface buttress plate
